# The Impact of Navigation in Lumbar Spine Surgery: A Study of Historical Aspects, Current Techniques and Future Directions

**DOI:** 10.3390/jcm13164663

**Published:** 2024-08-08

**Authors:** Ahmed Majid Heydar, Masato Tanaka, Shrinivas P. Prabhu, Tadashi Komatsubara, Shinya Arataki, Shogo Yashiro, Akihiro Kanamaru, Kazumasa Nanba, Hongfei Xiang, Huynh Kim Hieu

**Affiliations:** 1 Department of Orthopedic Surgery, Okayama Rosai Hospital, 1-10-25 Chikkomidorimachi, Okayama 702-8055, Japan; dr.a.heydr@gmail.com (A.M.H.); shriniprabhu62@gmail.com (S.P.P.); t.komatsubara1982@gmail.com (T.K.); araoyo@gmail.com (S.A.); m11099sy@jichi.ac.jp (S.Y.); akihiro198326@gmail.com (A.K.); sooseizi0402@yahoo.co.jp (K.N.); xianghf@qdu.edu.cn (H.X.); huynkimhieumd@gmail.com (H.K.H.); 2 Orthopedic and Traumatology Clinic, Memorial Bahçelievler Hospital, Bahçelievler Merkez, Adnan Kahveci Blv. No: 227, 34180 İstanbul, Turkey

**Keywords:** lumbar surgery, navigation, spine, image guidance

## Abstract

**Background/Objectives**: We sought to improve accuracy while minimizing radiation hazards, improving surgical outcomes, and preventing potential complications. Despite the increasing popularity of these systems, a limited number of papers have been published addressing the historical evolution, detailing the areas of use, and discussing the advantages and disadvantages, of this increasingly popular system in lumbar spine surgery. Our objective was to offer readers a concise overview of navigation system history in lumbar spine surgeries, the techniques involved, the advantages and disadvantages, and suggestions for future enhancements to the system. **Methods**: A comprehensive review of the literature was conducted, focusing on the development and implementation of navigation systems in lumbar spine surgeries. Our sources include PubMed-indexed peer-reviewed journals, clinical trial data, and case studies involving technologies such as computer-assisted surgery (CAS), image-guided surgery (IGS), and robotic-assisted systems. **Results**: To develop more practical, effective, and accurate navigation techniques for spine surgery, consistent advancements have been made over the past four decades. This technological progress began in the late 20th century and has since encompassed image-guided surgery, intraoperative imaging, advanced navigation combined with robotic assistance, and artificial intelligence. These technological advancements have significantly improved the accuracy of implant placement, reducing the risk of misplacement and related complications. Navigation has also been found to be particularly useful in tumor resection and minimally invasive surgery (MIS), where conventional anatomic landmarks are lacking or, in the case of MIS, not visible. Additionally, these innovations have led to shorter operative times, decreased radiation exposure for patients and surgical teams, and lower rates of reoperation. As navigation technology continues to evolve, future innovations are anticipated to further enhance the capabilities and accessibility of these systems, ultimately leading to improved patient outcomes in lumbar spine surgery. **Conclusions**: The initial limited utilization of navigation system in spine surgery has further expanded to encompass almost all fields of lumbar spine surgeries. As the cost-effectiveness and number of trained surgeons improve, a wider use of the system will be ensured so that the navigation system will be an indispensable tool in lumbar spine surgery. However, continued research and development, along with training programs for surgeons, are essential to fully realize the potential of these technologies in clinical practice.

## 1. Introduction

The advancement of navigation in spine surgery can be traced back to the inception of stereotactic techniques in neurosurgery during the early 20th century [1]. However, it was not until the latter part of the 20th century, when computer technology and sophisticated imaging modalities became available, that these techniques applied to spine surgery [2]. Navigation for treating deformities, degenerative diseases, trauma, and tumors of the lumbar spine to aid in decompression, pedicle screw insertion, interbody fusion, and bone resection has been widely used in recent years [3,4,5]. The intricate anatomical structure of the spine, in close proximity to vital structures such as the spinal cord and major blood vessels, makes surgical procedures such as decompression and implant placement particularly challenging. The implementation of accurate navigation during these procedures is essential to improve precision, avoid iatrogenic neurovascular injuries, and improve surgical outcomes [6]. This method employs advanced imaging techniques and computer-assisted systems to guide surgeons in real time, ensuring the precise placement of instruments and implants [7].

The benefits of navigation in spine surgery are significant and multifaceted, in addition to improving implant positioning accuracy and decompression adequacy and reducing radiation exposure, when compared to fluoroscopy. The consequent minimization of the related risks is especially important for surgeons and medical staff because of their constant presence in these operations [8]. Additionally, better clinical outcomes, including a reduced rate of revision surgeries, have been reported [9]. However, navigation in lumbar spine surgery is not without its challenging aspects, such as high cost, steep learning curve for surgeons and operating room staff, and chances of inaccurate tracking leading to system errors [10]. Ongoing research in navigation includes integration with robotics, augmented reality, and even the use of artificial intelligence to improve current navigation systems [11]. Upon reviewing the literature, a limited number of papers were found that addressed the historical evolution, detailed the areas of use, and discussed the advantages and disadvantages of this increasingly popular system in lumbar spine surgery. Therefore, our objective was to offer readers a concise overview of navigation history, the techniques involved, the advantages and disadvantages, and suggestions for future enhancements to the system.

## 2. Materials and Methods

A comprehensive review of the literature was conducted, focusing on the development and implementation of navigation systems in lumbar spine surgeries. Our sources include PubMed-indexed peer-reviewed journals, clinical trial data, and case studies involving technologies such as computer-assisted surgery (CAS), image-guided surgery (IGS), and robotic-assisted systems. The keywords “navigation”, “pedicle”, “fluoroscopy”, “CT”, “3D”, and “spine” were used while searching the databases. We focused on papers published after 2000, when the use of CT-guided navigation became prevalent. We excluded case reports, technical note review articles, and publications with an impact factor less than 1.

## 3. Results

The database search yielded valuable information about the historical evolution of navigation systems in spinal surgery, current implementation, and surgical outcomes. We summarized them as follows.

### 3.1. Historical Overview of Navigation in Lumbar Spine Surgery

The evolution of navigation in spine surgery has involved technological advancements and clinical refinements. This progression has significantly improved the precision and outcomes of spinal procedures. Below is a historical overview highlighting key milestones.

#### 3.1.1. In the 1980s (The Initial Concept of Surgical Navigation)

This concept emerged in the late 20th century, with early systems focusing on brain surgery [12]. These systems utilize preoperative imaging and basic tracking technologies to guide surgeons during procedures. The adaptation of these principles to spine surgery followed soon after, leveraging the detailed anatomical visualization provided by CT and MRI scans.

#### 3.1.2. In the Early 1990s (Emergence of Image-Guided Surgery)

In this era, significant advancements in intraoperative imaging, particularly the integration of fluoroscopy with navigation systems, have been made [13,14]. Fluoroscopy-based navigation allows real-time imaging during surgery, improving the accuracy of instrument placement [15]. However, this method also poses challenges such as limited image quality and increased radiation exposure [16]. In the 1990s, the field of image-guided surgery for spine surgery was still developing, with initial advances heavily reliant on navigation techniques that were primarily designed for cranial interventions [3]. One of two registration types was employed in cranial navigation: fiducial-based or surface-based registration. Fiducial-based registration methods depend on localizers positioned on the surface of skin or bone before a preoperative CT scan or MRI is obtained. In contrast, surface-based registration utilizes a localizer to digitalize the head or vertebra surface, which is then compared to a surface model obtained from CT or MR images [3]. For both types of registration, issues such as time for registration, accuracy [17], and invasiveness [18] made these techniques less reliable in practice.

#### 3.1.3. In the Late 1990s (Introduction of Computer-Assisted Surgery)

In clinical practice, fluoroscopic imaging-based navigation technologies are available [14]. Independently, the Medtronic, Medivision, and Brain Lab developed virtual fluoroscopic navigation systems, focusing on mobile and image intensifier C-arms, which are the dominant intraoperative imaging methods used in operating rooms [14]. However, these technologies have limitations, such as low image quality and radiation exposure. Fluoroscopic-assisted navigation enables automatic spinal registration, improved image quality, and decreased radiologic exposure to surgical personnel [13]. Despite these advantages, the lack of 3D views, especially axial images, during navigation is regarded as a significant drawback [13]. The late 1990s and early 2000s marked the introduction of computer-assisted surgery (CAS) in spine surgery. CAS systems combined preoperative 3D imaging with intraoperative navigation, enhancing the precision of pedicle screw placement. Early studies demonstrated improved accuracy and reduced complication rates compared to conventional methods [19].

Additionally, in this period of time, intraoperative usage of ultrasonography as a real time imaging tool gained popularity especially during the reduction and stabilization of burst fractures; they were used for evaluation of backward displacement of the spinal posterior elements and in checking spinal canal clearance [20]. Intraoperative ultrasonography was also used to help surgical planning of intradural spinal lesions by locating the lesion, determining its dimensions, and optimizing the incision of dura and myelotomy so that the shortest way to the lesion can be pursued.

#### 3.1.4. In the Early 2000s (Advances in 3D Navigation Systems)

The early 2000s witnessed the development of more sophisticated 3D navigation systems. These systems utilized high-resolution CT scans to create detailed 3D models of the spine, which were then used intraoperatively to guide surgical instruments. This period also saw the advent of image-guided surgery (IGS), which further improved surgical accuracy and outcomes [17]. Tomoscan (Philips Medical System) was one of the earliest intraoperative mobile CT systems used for successful spinal navigation [19]. Limitations included a closed gantry and a relatively small bore size. Thereafter, in 2002, Iso-C was released by Siemens as the first C-arm that can perform three-dimensional reconstruction of the spinal column [21]. In the same time frame, the integration of preoperative CT data with anteroposterior and lateral fluoroscopic images facilitated a semiautomatic registration process for the three-dimensional data, enabling access to these data in the operating room without the need for the time-consuming and challenging paired-point registration that is typically used with cranial image-guided surgery [3].

During the same period, an O-arm^®^ Imaging System (Medtronic) was used. This was a result of combining high-quality CT images and the simple automatic registration of the Iso-C (Siemens) system. The modifiable gantry that enables side access of patients with advanced autoregistration and provides higher image quality [22]. In addition, the reduction in time necessary for the registration process is also considered to be among the most important advantages [23]. The implementation of automatically registered three-dimensional imaging in navigated spine surgery and has contributed to a proliferation of navigated surgical instruments that enhance minimally invasive surgeries [3].

#### 3.1.5. In the 2010s (The Integration of Navigation with Robotic Systems)

The most significant achievement in this decade was the integration of navigation with robotic systems, which represents a major improvement in spine surgery technology. Robotic-assisted systems provide enhanced precision and control, allowing for more complex and minimally invasive procedures. Early adopters reported positive outcomes, including reduced surgical times and improved screw placement accuracy [24].

In recent years, augmented reality (AR) and artificial intelligence (AI) have emerged for spine surgery navigation. AR systems offer immersive visualization, overlaying critical information directly onto the surgical field. AI, on the other hand, assists in preoperative planning and intraoperative decision-making, providing data-driven insights to enhance surgical precision [25]. Table 1 summarizes the significant milestones in the development and integration of navigation technologies, demonstrating the evolution from basic imaging techniques to sophisticated AI and robotic-assisted systems.

### 3.2. Current Navigation Systems

Currently, the most frequently used intraoperative imaging systems are O-arm™ (Medtronic©, Minneapolis, MN, USA), Ziehm Vision RFD 3D™ (Ziehm Imaging©, Orlando, FL, USA), and Airo^®^ (Brainlab©, Feldkirchen, Germany). These imaging platforms are typically compatible and work in coordination with navigation instrumentation software, such as StealthStation S8 (Medtronic©, Minneapolis, MN, USA), Spine Mask© Version 2.2 (Stryker©, Kalamazoo, MI, USA) and the 7D Surgical System Version 1 (7D Surgical©, Toronto, ON, Canada).

The FDA approved the Airo^®^ mobile intraoperative CT in 2013, and the images obtained by this machine were analyzed by the specified Brainlab© software Version 3.1. These platforms have a large scanner that can provide a sufficient field for larger patients. The ability of the imaging system to rotate helps provide comfortable intubation and intravascular line placement by anesthesia staff. Being portable, the ability to move them to other operating rooms and the possibility of performing two navigated spine surgeries on the same day are also considered advantages of the imaging system.

The O-arm™ is another widely employed intraoperative imaging machine for providing 2D/3D imaging. These machines have a C-shaped gantry resembling a C-arm but have the ability to close around the surgical table and therefore provide 360° images. They are compatible with the StealthStation S8 navigation software manufactured by the same company. Alternatively, the Ziehm Vision™ platform also appears to be a C-arm but can obtain intraoperative high-quality images by rotating automatically around the surgical table. It has another advantage of being capable of coordinating with any available navigation software.

### 3.3. Steps of Applied Navigation in Lumbar Spine Surgery

The steps necessary for navigation in spine surgery are generally similar for all navigation systems. Here, we summarize the protocols that are followed in our institution concerning preoperative planning, intraoperative decision-making, and postoperative assessment.

#### 3.3.1. Preoperative Planning

**Figure 1:**
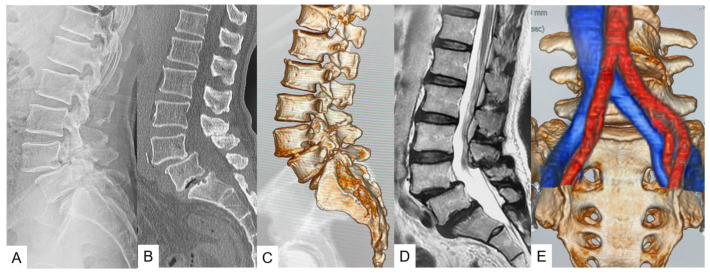
Preoperative images. (**A**) Lateral lumbar neutral radiogram, (**B**) mid-sagittal reconstruction CT, (**C**) 3-D CT, (**D**) T2-weighted mid-sagittal MR imaging, (**E**) Ct-MRI fusion image. For oblique lateral interbody fusion at L5-S1 (OLIF51), evaluation of the vascular window is very important.

*Imaging*: High-resolution CT and MRI scans were taken preoperatively to create a 3D model of the patient’s spine (Figure 1).

*Software Analysis*: The scans are uploaded into navigation software to plan the optimal surgical approach and implant placement.

#### 3.3.2. Patient Positioning and Registration

*Positioning*: The patient is positioned on the operating table, typically prone or lateral to match the preoperative image.

**Figure 2:**
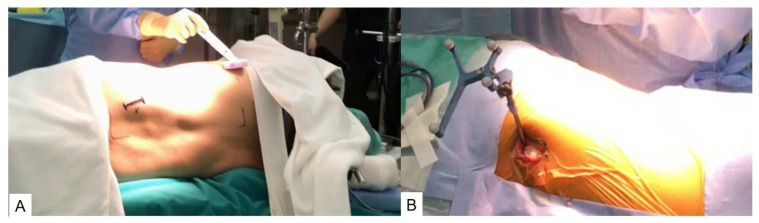
Patient position and dynamic reference frame. (**A**) Lateral decubitus position for OLIF. (**B**) A dynamic reference frame is inserted into the sacroiliac joint.

Reference frame placement: The success of navigated spine interventions depends partly on the reliability of instrument localization and patient tracking relative to a common reference coordinate system. Typically, a dynamic reference frame (DRF) is affixed to the patient to facilitate accurate tracking. The placement of the DRF is crucial, as it can impact overall accuracy based on its distance from the treatment site [26]. The surgeon must choose a position that does not obstruct their line of sight with the optical localizer while maintaining a clear path for instruments [27]. In the upper lumbar spine, the DRF is commonly clamped to the spinous process [28]. In the lower lumbar/sacral spine, the DRF is sometimes attached with a pin to the posterior superior iliac spine or sacroiliac joint [27,29]. Regardless of the attachment site, the DRF must remain rigid throughout the procedure. Inaccuracies can occur due to improper fixation of the DRF [30] or if it is too far from the area of intervention [31] (Figure 2).

#### 3.3.3. Intraoperative Navigation

Navigation inaccuracies can result from movement of the DRF relative to initial anatomic localization during surgery, inaccurate calibration of the image volume concerning the imager tracking array, or distortion of the anatomy due to surgical intervention [32]. The use of electromagnetic tracking or other non-line-of-sight tracking technologies would allow for tracking multiple references and has been studied for spinal applications [33].

**Figure 3:**
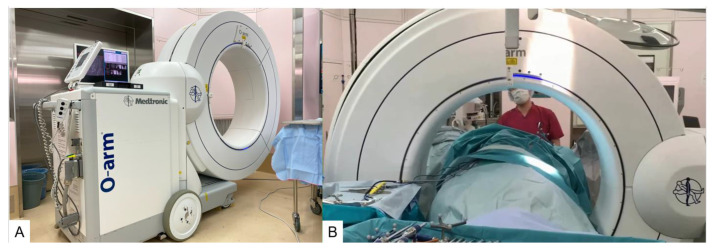
O-arm. (**A**): The appearance of O-arm, (**B**): Intraoperative use of O-arm.

Real-Time Imaging: Intraoperative CT or fluoroscopy is used to continuously update the navigation system with real-time images (Figure 3).

*Guidance*: The surgeon uses the navigation system’s real-time feedback to guide instruments and place implants with high precision following the preoperative plan.

#### 3.3.4. Registration, Verification, and Adjustment

**Figure 4:**
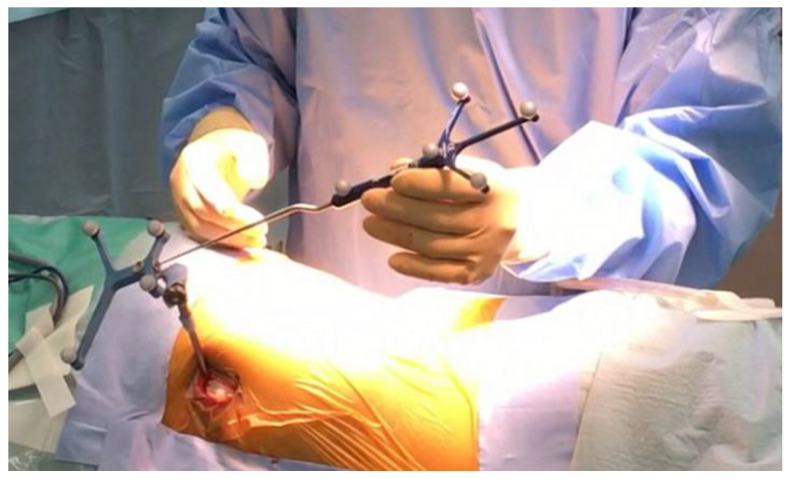
Registration of ball tip pointer.

*Registration*: Every instrument should be registered before usage (Figure 4).

*Accuracy Check*: The final positions of the implants were verified using intraoperative imaging to ensure proper placement.

*Adjustments*: If necessary, adjustments are made based on real-time feedback to correct any deviations from the planned path.

#### 3.3.5. Navigated Instruments

**Figure 5:**
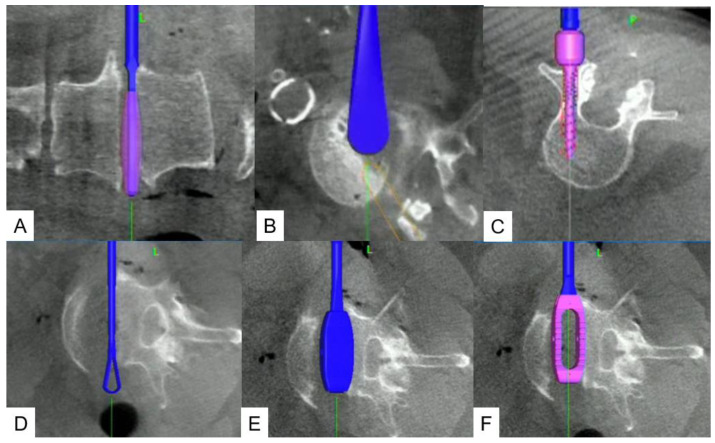
Various kinds of navigated instruments: (**A**) navigated shaver, (**B**) cobb, (**C**) pedicle screw, (**D**) curet, (**E**) cage trial, (**F**) cage.

*Navigated Instruments*: Various kinds of instruments are available, such as a shaver, Cobb elevator, curet, trial, cage, and pedicle screw (Figure 5).

#### 3.3.6. Postoperative Assessment

**Figure 6:**
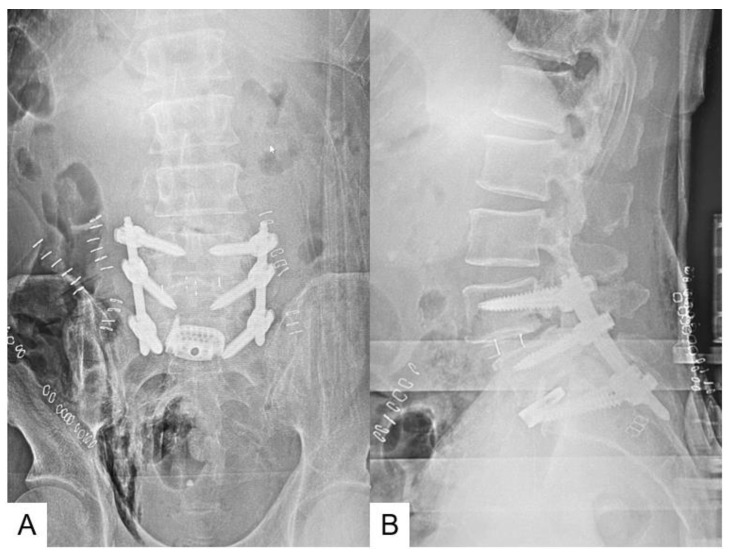
Postoperative images. (**A**): Anteroposterior lumbar radiogram, (**B**): Lateral lumbar radiogram.

The final images of the patients were taken inside the operating room before the patient was placed on the surgical table. Postoperative CT or X-rays were used to confirm the correct placement of the implants and the success of the surgery (Figure 6). Rivkin et al., discussed this postoperative confirmation of accurate implant placement by intraoperative three-dimensional imaging [34]. This approach ensures that the ultimate verification before the operation is terminated, provided that the desired surgical intervention has been performed successfully without complications. This enables surgeons to adjust any improper implant placement immediately without requiring a revision operation. This valuable tool is especially beneficial in early experience, as it may enhance patient outcomes and boost the confidence of newly introduced surgeons in navigation systems.

### 3.4. The Role of Navigation Systems in Lumbar Spine Surgery and Their Effectiveness

The integration of navigation systems in lumbar spine surgeries has a profound impact on lumbar spinal surgeries. For the first instance, the utilization of navigation systems was mainly focused on the accuracy and safety of implant placement (especially for pedicle screws), but with the innovation of navigated instruments such as osteotomes, drills, and burrs, the use of this technology was further expanded in such a way that it entered all fields of lumbar spinal surgery. Studies have shown that computer-assisted navigation systems provide real-time 3D imaging, enhancing surgeons’ ability to navigate and intervene in complex anatomical procedures while avoiding injury to neurovascular structures. These technologies have led to decreased operative times, reduced radiation exposure for both patients and surgical teams, and lower rates of reoperation.

#### 3.4.1. Role of Navigation in Lumbar Pedicle Screw Placement

Dural tears, spinal cord complications, and nerve damage complications were the main complications of free-hand pedicle screw placement. A high incidence of free-hand technique pedicle screw placement, which may exceed 35% of placed pedicle screws, has been reported in several studies [35]. Fluoroscopy-based pedicle screw placement also has a high rate of misplacement, which may reach 22% [36]. Therefore, every effort was concentrated on the development of new techniques to reduce the possibility of misplacement and related complications. The primary goal of computer-assisted navigation system development was to increase the accuracy and safety of pedicle screw placement. In a randomized prospective study, Yu et al., compared the accuracy of pedicle screw placement in the lumbar spine of 401 patients between the freehand technique and computer-assisted navigation system; the excellent rate of pedicle screw position placed with navigated system was 95% versus 84% placed with freehand technique, and they concluded that these systems are effective at preventing pedicle screw misplacement and reducing the overall operation duration [37]. The superiority of screw placement accuracy achieved by navigation systems over fluoroscopy base placement was demonstrated by another study [38]. In the same study, the authors showed that the screws placed by the navigation system were significantly larger and had a significantly lower revision rate.

Houten et al., in a comparative retrospective study, investigated the efficacy of fluoroscopy-guided navigation versus O-arm-guided navigation in percutaneous lumbar procedures. Their findings revealed a perforation rate of 12.8% in 141 screws placed during fluoroscopy-guided procedures, while O-arm-guided procedures had a rate of 3% of placed 205 screws. Additionally, the use of O-arm navigation was found to reduce the procedure duration by an average of 20 min [39]. In addition, they found that the use of the O-arm shortened the length of the procedures by a mean of 20 min. By comparing preoperative and intraoperative CT-based navigation accuracy, Wood et al., reported that intraoperative 3D imaging was more precise (1.6% compared to 6.4%) and that using EMG monitoring of lumbar nerve roots during screw insertion further improved the accuracy [40].

#### 3.4.2. Role of Navigation in Lumbar Interbody Implant Placement

Interbody fusion consists of removing the intervertebral disc and inserting a graft or implant and is associated with reduced rates of postoperative pseudoarthrosis [41]. It was originally described by Briggs and Milligan in 1944, who used a posterior approach to lumbar interbody fusion [42] and developed it as an alternative to posterolateral fusion techniques. Lumbar interbody fusion is used to treat various spinal pathologies resulting from degenerative disease, deformity, trauma, infection, and neoplastic disorders. Eventually, many other approaches have evolved, including anterior, lateral, oblique, and transforaminal approaches.

In lumbar interbody fusion, appropriate cage placement is crucial to ensure perpendicular alignment to the intervertebral disc space and to avoid disruption of the anterior longitudinal ligaments and encroachment upon the spinal canal. Biplanar fluoroscopy can be used to achieve cage placement, but it can be cumbersome and can lead to increased radiation exposure [43]. Navigation can be used in interbody implant placement procedures to avoid the need for intraoperative fluoroscopy and increased radiation exposure. In a recent retrospective cohort study, 87 patients were compared in respect of accuracy of cage placement and radiation exposure in lateral lumbar interbody fusion between intraoperative navigation and conventional fluoroscopy; the authors demonstrated that the accuracy of implant placement and radiation exposure in navigation-assisted lateral lumbar interbody fusion were comparable to those obtained using fluoroscopy, with the sole benefit of reducing the radiation dose to the surgeon and surgical team [44]. However, in their retrospective radiological analysis of 127 lateral cages in 75 consecutive patients comparing the intraoperative and postoperative measurement of lateral cage placement using fluoroscopy, Chung et al. [45] concluded that intraoperative C-arm images underestimate midline deviation and obliquity of the cage. On the other hand, studies in the literature have reported that the accuracy of cage placement with intraoperative navigation may reach 98.3% [46].

**Figure 7:**
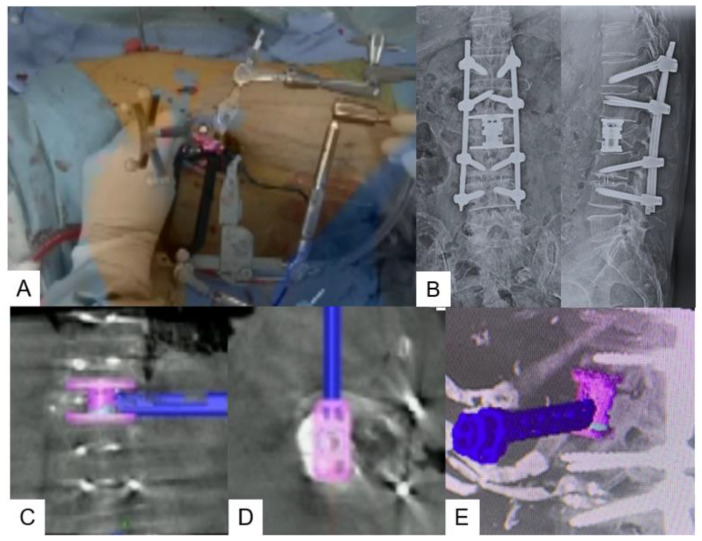
The expandable vertebral cage was navigated. (**A**) Intraoperative image, (**B**) postoperative radiograms, (**C**) coronal image, (**D**) axial image, (**E**) sagittal image.

Cage placement could be difficult following corpectomy due to the lordotic alignment between the proximal and distal vertebra, especially in the segment between L4 and the sacrum [47]. However, by applying a navigated cage, the appropriate cage positioning could be achieved with the aid of live feedback trials, which frequently allow for the insertion of a larger cage. Moreover, navigated expandable vertebral cages have a significantly lower malposition rate than those placed under conventional fluoroscopy [48] (Figure 7). However, performing these operations without the use of fluoroscopy requires a steep learning curve, and an intraoperative complication could be catastrophic [5].

Traditionally, changing the patient’s position during surgery is unnecessary in OLIF operations since the cage is placed in the lateral decubitus position, while the percutaneous pedicle screw is placed in the prone position. With the aid of navigation, both procedures can be performed effectively and safely in a single lateral position, reducing surgical duration, blood loss, and postoperative rehabilitation time [49].

#### 3.4.3. Role of Navigation in Minimally Invasive Lumbar Decompression

**Figure 8:**
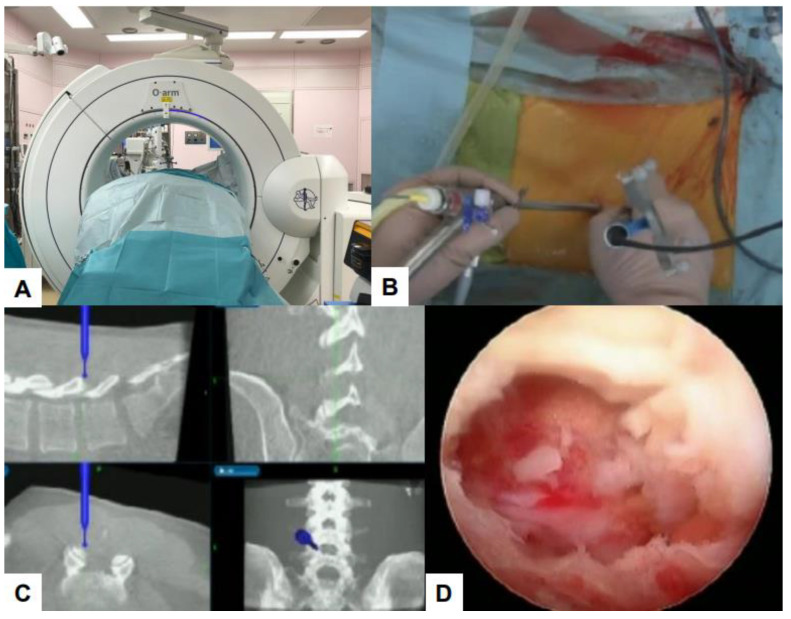
C: arm-free UBE, (**A**) O arm, (**B**) intraoperative image, (**C**) navigation monitor, (**D**) endoscopy image [52].

As the minimally invasive nature of lumbar spine surgery increases, the need for C-arm imaging also increases. Although radiation exposure poses a relatively minor risk to treated patients, it can accumulate and pose a danger to surgeons and their teams over time, who are repeatedly exposed to such radiation throughout their careers [50]. To overcome these hazards in lumbar spine surgery, navigation techniques were implemented to optimize the trajectory for endoscope and instrument insertion and decompression [51] (Figure 8). Navigation systems, such as CT-assisted navigation, that utilize real-time images have been shown to enhance surgical accuracy and reduce the complication rate [52]. For instance, the surgical management of foraminal and extraforaminal stenosis at the L5-S1 level can be challenging due to anatomical characteristics and degenerative changes that make the operating window extremely narrow. Navigation can not only provide adequate decompression but also minimize excess bone removal from the facet joint, thereby significantly preventing potential iatrogenic instability and accelerated degeneration [23].

#### 3.4.4. Role of Navigation in Lumbar Spinal Tumors and Other Cystic Lesions

**Figure 9:**
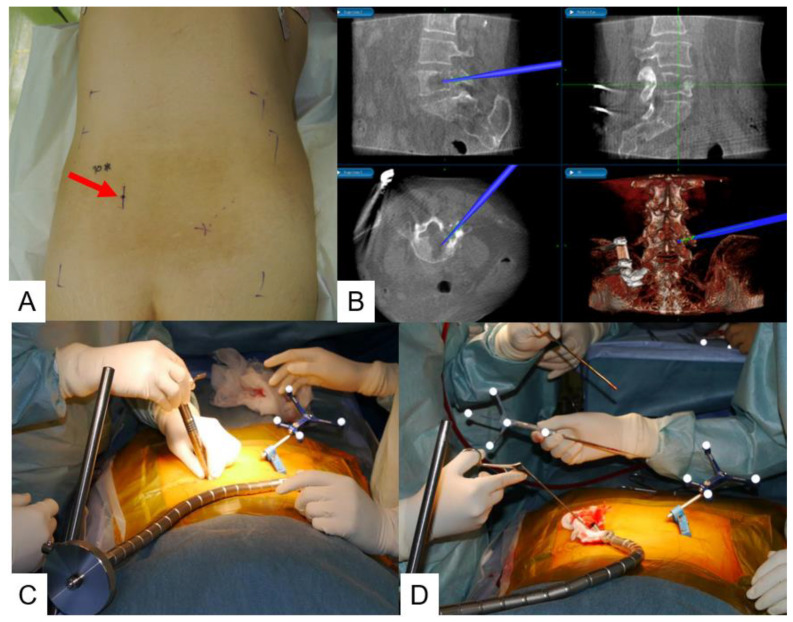
Male, 19, L5 osteosarcoma, biopsy, (**A**) 1.6 cm skin incision, (**B**) navigation image, (**C**) sequential dilation, (**D**) navigation biopsy. A red arrow indicates a skin incision. A blue navigated probe is located in the center of the tumor.

Navigation systems are increasingly being used to treat spinal tumors for both diagnostic and therapeutic purposes. Navigation-guided biopsy is an effective technique for obtaining sufficient tissue samples from various spine pathologies and may be a better alternative to CT-guided and open biopsy (Figure 9). High radiation exposure, insufficient samples, and injuries to major neurovascular structures can be overcome by navigation systems [53]. The therapeutic uses of navigation systems can be divided into two main categories: tumor resection in addition to reconstruction and stabilization. The primary objective of surgery for primary spinal column tumors is complete tumor resection with clean surgical margins, which necessitates careful planning of the exposure, precise osteotomy trajectories, and visualization of complex, three-dimensional anatomy that may be distorted by the tumor. To ensure adequate bony resection while navigating challenging anatomy, image guidance is essential for maintaining the osteotomy according to the preoperative plan, which can be critical for maintaining tumor margin integrity [54]. This can be achieved by intraoperative CT navigation, but some studies have reported using a combination of MR and CT images in the treatment of sacral chordomas [55].

In addition to lumbar spinal tumors, navigation systems could be used effectively in the treatment of benign cystic lesion of the lumbar spinal cord. The cystic dilation of ventriculus terminalis, which is an embryological remnant lined by ependymal cells [56], was successfully and effectively evacuated using real-time magnetic resonance navigation [57]. Also, Bonsanto et al., were able to localize syrinx in seven patient using 3D ultrasound navigation and concluded that such navigation was helpful and more practical for the determination of orientation and lesion border [58].

In contrast, spinal cord and nerve root decompression are the objectives of spinal metastatic tumor surgery. Recently, separation surgery has been recommended for optimal adjuvant radiation therapy. Generally, total tumor excision cannot be achieved in these operations [59]. The objective of separation surgery is to create a space between the tumor and the spinal dura, which allows for postoperative delivery of adequate doses of spinal stereotactic radiosurgery. This provides dependable metastatic control, regardless of the size and histology of the tumor [60]. In cases where direct visualization is not possible, navigation guidance becomes the most reliable way of visualizing the anterior epidural space [61].

Spinal column reconstruction following tumor resection and spinal fixation following instability in oncologic patients are separate topics within the field of spinal tumors. Oncologic patients usually have osteoporotic bone quality due to various factors, such as the tumor itself, chemotherapy, radiation, malnutrition, and other age-related comorbidities. As a result, fusion is difficult to achieve, and stabilization is primarily obtained from spinal instrumentation with or without cement augmentation. Therefore, navigation system stabilization could be achieved with less invasive procedures in these debilitated patients [28].

#### 3.4.5. Role of Navigation in Spinal Osteotomy and Deformity Correction

Navigation can play an essential role in improving the accuracy of screw and interbody cage placement, as well as planning three-column osteotomies. By providing a three-dimensional visualization of the extent and trajectory of osteotomy, navigation can help mitigate the risks associated with spinal deformity surgery. This can be particularly useful for pedicle subtraction osteotomy and vertebral column resection, as it can optimize the accuracy of bone cuts and actualize the preoperative plan of the osteotomies [62]. The resultant undesired gaps following asymmetric osteotomies increase the risk of pseudoarthrosis, and inadequate osteotomies lead to a limited amount of correction that negatively affects surgical and radiological outcomes [63]. Additionally, studies have shown that navigated lateral osteotomy of a fused mass in an adult spinal deformity can be performed safely and effectively [64]. Navigation is also useful in hemivertebrectomy for congenital scoliotic and kyphoscoliotic correction operations, as demonstrated by Takahashi et al. [65]. In their case series of eight patients, their mean kyphotic and scoliotic curve of 55.8° and 50.0° were corrected to 23.2° and 31.6°, respectively. They reported the use of navigation to confirm the position of the vertebra, plan the osteotomies, and localize the spinal cord and aorta in real time, minimizing potential neurovascular complications. Finally, recent reports have demonstrated the effective and safe use of navigation in the anterior correction of lumbar scoliosis [66].

#### 3.4.6. Role of Navigation in Lumbar Spine Fractures and Spinal Spondylolisthesis

Owing to the benefits of minimally invasive surgery with percutaneous pedicle screws in the treatment of lumbar fractures compared to open procedures [67,68], this technique has become the standard method for stabilizing thoracolumbar burst fractures; however, prolonged intraoperative C-arm usage and its hazards have become concerns for operating surgeons and operation room staff [5]. This disadvantage was overcome by the use of navigation [65]. Another advantage of navigation systems is overcoming the difficulty resulting from complex anatomy and unpredictable trajectories, thus improving the accuracy of implant placement and reducing the necessity of revision due to screw malposition [69]. Similarly, a mini-open lateral approach corpectomy for treating thoracolumbar fractures significantly improved surgical outcomes [70]. Yu et al., in a retrospective study, used intraoperative navigation for minimally invasive corpectomy and stabilization of unstable thoracolumbar burst fractures to reduce radiation exposure during surgery. With a total of 20 patients, they compared the surgical outcomes between intraoperative fluoroscopic and navigation guidance techniques and reported that clinical outcomes were comparable in both groups, with decreased radiation exposure to the operating surgeon [67] and operating room staff in surgeries that utilized navigation [71].

**Figure 10:**
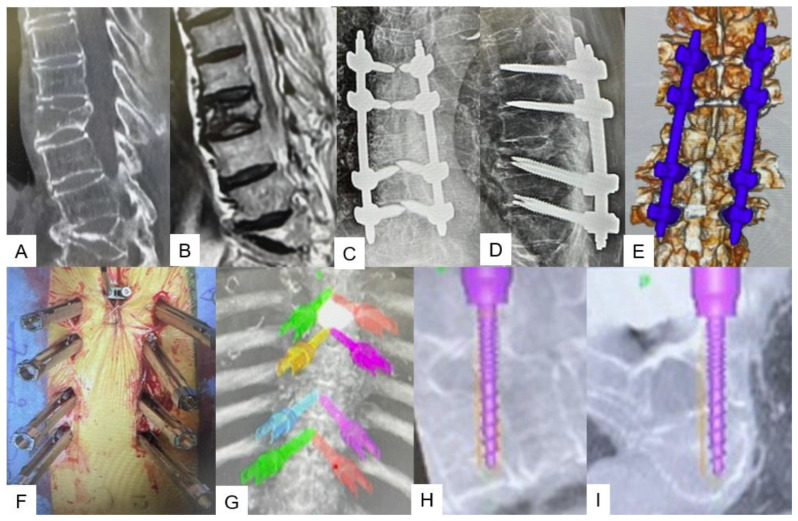
T9 extension fracture. (**A**): Preoperative midsagittal reconstruction CT, (**B**): Preoperative midsagittal T2 weighted MRI, (**C**): Postoperative anteroposterior radiogram, (**D**): Postoperative lateral radiogram, (**E**): 3D CT, (**F**): intraoperative image, (**G**): 3D image of navigation, (**H**): Sagittal image of transdiscal screw, (**I**): Axial image of transdiscal screw.

Transdiscal pedicle screw fixation was originally used by Abdu et al., for rigid fixation of spondylolisthesis [72], and subsequently, biomechanical studies demonstrated that these screws have a fixation strength of 1.6 to 1.8 greater than traditional pedicle fixation strength [73]. However, the technical challenge of the unique trajectory and difficulty in obtaining good-quality intraoperative fluoroscopic images limits its applicability [74]. With the advancement of the intraoperative navigation system, transdiscal pedicle placement has become more feasible, and good clinical and radiological outcomes with no intraoperative or postoperative complications have been reported for in situ fusion using transdiscal pedicle screws [75,76]. Owing to the ease of placement of transdiscal pedicle screws, their indications have expanded to include complicated thoracolumbar vertebral pathological fractures. In osteoporotic vertebrae, pedicles become enlarged due to bone remodeling, resulting in less strong pedicle screw fixation, which could represent a better option for fixation of these fractures [77]. Other candidates for this technique are thoracolumbar fractures in diffuse idiopathic skeletal hyperostosis patients (DISH) due to diminished traditional screw fixation strength [78,79] and increased stress concentration of spinal instrumentation around the fracture site [46] on the fracture site because of the long lever arm of the ankylosed spine, which may lead to screw pullout and loosening and consequent pseudoarthrosis [75] (Figure 10). Ikuma et al. [80], in a retrospective case series of 13 patients, reported stable surgical outcomes in elderly patients with DISH-associated thoracolumbar spinal fractures treated with navigation-assisted transdiscal pedicle screws, and they demonstrated that transdiscal screws are stronger anchors than traditional pedicle screws [80].

Patients with certain rheumatological conditions, such as ankylosing spondylosis due to osteoporosis, spinal fusion, and deformity, are more susceptible to spinal fractures with a low energy impact [81]. As these fractures are generally unstable, approximately 67% of them have neurological deficits, and secondary frequent deterioration is documented [81]; thus, surgical fixation should be promptly performed. However, due to the nature of the disease, the anatomical landmarks are distorted, and therefore, a risk of nerve injury arises during screw insertion. Studies have demonstrated that the use of a navigation system in these patients reduces surgical time, minimizes blood loss, and enhances the accuracy of implant placement [82,83]. Furthermore, a recent retrospectively analyzed case series of 16 patients reported the safety and effectiveness of navigation-assisted posterior wedge osteotomy in the treatment of ankylosing patients with thoracolumbar fractures, which decompressed the spinal canal, stabilized the fracture, and significantly corrected kyphosis [84].

## 4. Discussion

Although the application of navigation systems in lumbar spine surgery represents a substantial advancement in the field with significantly improved outcomes, these systems are not devoid of disadvantages and challenges, necessitating a balanced discussion. Modern spine surgery navigation systems can be divided into three main categories [85]. 1. Optical Tracking Systems: These systems use infrared cameras and reflective markers attached to surgical instruments and the patient to track movements in real time. Examples include the StealthStation by Medtronic and the Brainlab navigation system. 2. Electromagnetic Tracking Systems: These systems utilize electromagnetic fields to track instruments and the patient. They are particularly useful in procedures where line-of-sight can be obstructed. 3. Intraoperative Imaging Systems: Incorporating real-time imaging, such as intraoperative CT or MRI scans, allows for continuous updates and adjustments during surgery, increasing accuracy. In the current review, we attempt to summarize the advantages, disadvantages, and future directions of these systems in the following points:

### 4.1. Advantages of Navigation in Spine Surgery

A study conducted by Virk et al., highlighted the significant role that image-guided navigation plays during spinal surgery. This method enhances the visualization of both bony and soft tissues by providing a larger area through a smaller surgical dissection. During the procedure, computer-based navigation systems offer guidance to MIS surgeons for instrumentation and non-instrumented surgeries [86]. These systems are beneficial for locating areas that need decompression, assessing the adequacy of decompression, and ensuring instrumentation accuracy. Using a reference frame and cross-sectional imaging, these systems create a detailed interactive image of the patient’s anatomy in real time. Real-time feedback and guidance allow surgeons to adjust their techniques during surgery, minimizing errors and improving overall surgical outcomes [2].

Providing real-time, precise anatomical visualization enhances patient safety. Navigation systems help in avoiding critical structures such as nerves and blood vessel injuries, with a consequent reduction in intraoperative and postoperative complications [87]. Khanna et al., in a retrospective study, compared the operative time of 70 one-level lumbar instrumentations assisted by navigation with the corresponding 63 freehand-placed instrumentations; the results showed shorter surgery times and less blood loss via navigation systems, which also contribute to further patient safety enhancement [88]. As a result, the use of navigation has been associated with better clinical outcomes, including lower rates of reoperation and fewer postoperative complications [89]. Patients benefit from improved recovery times and overall satisfaction [90]. Increased surgical efficiency can lead to shorter operative times and hospital stays, reducing healthcare costs in the long term [89].

Over the past 30 years, advancements in the techniques of minimally invasive surgery have led to an increased reliance on fluoroscopic imaging [91,92,93], exposing both patients and surgical teams to considerable amounts of radiation. This increased radiation exposure to surgeons and staff has become a significant concern that must be addressed. Navigation systems are one of the solutions that can reduce the dependency on fluoroscopy, lower radiation exposure, and reduce potential hazards [94]. The radiation exposure measurement of a surgical team using intraoperative navigation showed values below the occupational exposure limits set by the International Commission on Radiological Protection [95]. In navigated surgeries, during intraoperative imaging, surgeons and staff can be protected by a radiation shield or temporarily leave the operating room, which minimally affects the normal workflow [96]. In contrast to fluoroscopic procedures, surgeon exposure to radiation is more common because surgeons remain close to the radiation source [97].

While intraoperative three-dimensional imaging results in greater radiation exposure to patients than does conventional fluoroscopic procedures, the improved surgical outcomes may offset the added dose by eliminating the need for preoperative or postoperative CT scans [3]. Researchers have evaluated the impact of various O-arm dose settings using phantoms and pig spines [98]. They found that the radiation dose for the O-arm could be decreased by five to thirteen times without compromising the visualization of important anatomical landmarks necessary for pedicle hardware placement. This is particularly significant for younger patients or those with a low body mass index. Advanced reconstruction algorithms can further reduce patient dose. Filtered back projection (FBP) is the most prevalent technique due to its simplicity and rapidity. More recent advanced algorithms, such as algebraic or iterative reconstruction techniques, boast several advantages over FBP methods, such as the ability to reconstruct volumes using fewer or noisy images [99]. Nevertheless, employing these advanced algorithms demands increased computational resources, including bandwidth or reconstruction time, which may not always be accessible.

### 4.2. Disadvantages of Navigation in Spine Surgery

Navigation, however, has its own set of challenges, which may limit its use. These include cost-effectiveness, a steep learning curve, and the need for highly qualified operation room staff.

1. High Cost: The initial investment in navigation systems is substantial, which can be a barrier for many healthcare facilities, especially those in resource-limited settings. Maintenance and updates also add to the cost [100]. The economic burden extends to training personnel and integrating these systems into existing workflows [101]. Despite advancements in the field of navigated spine surgery over the past two decades and the widespread availability of navigation systems, a mere 9% of surgeons consistently employ these systems [1].
2. Learning Curve: Surgeons and operating room staff need to undergo extensive training to become proficient in using navigation systems. The learning curve can initially lead to longer operative times and increased complexity in case management [102]. Proficiency requires consistent use and practice, which can be challenging in low-volume centers [103]. The surgeons reported that their first experience did not comply with their expectations. As they progressed along the learning curve, the team identified several critical factors for success, such as the optimal camera position, reference frame attachment location and orientation, recognition of possible causes of errors that the system possesses, and proper steps in the surgical workflow [34]. As they became competent with these parameters, their ability to efficiently place screws significantly improved.

Using a CT scan, Sclafani et al., in a cadaveric study, demonstrated that preciseness was constant along the learning curve compared to an inverse correlation between speed and preciseness with two-dimensional C-arm images alone [29]. Experience with both techniques may increase the duration of screw placement. Some researchers have documented a learning curve associated with navigation in terms of the pedicle screw placement time and accuracy [104]. They discovered that the steepest learning curve was linked to the insertion of the pedicle screws in the thoracic vertebrae, mainly due to the smaller diameter of the pedicles. This learning curve has been extensively documented, and some experts have even referred to spinal navigation with intraoperative imaging as a “paradigm shift” [34].

Rahmathulla et al. [28] reported that improving the efficiency of the navigation system in a navigated spine operation requires surgeons and room staff to be harmonious throughout the surgery, and issues such as the positioning of the intraoperative imaging machine and other navigation equipment, placement of the navigation reference, and patient draping and neuromonitoring must be considered. The tracking technology used by navigation systems, either through electromagnetic sensors or optical cameras, has limited working abilities and different tracking accuracies depending on the angle and distance between the transmitter and detector. When utilizing these technologies, it is essential to follow the manufacturer’s instructions for placing the tracking systems and navigated instruments during the surgical procedure.

### 4.3. Future of Navigation in Spine Surgery

The future of navigation in spine surgery is poised for remarkable advancements, driven by ongoing technological innovations and clinical research. These advancements promise to further enhance surgical precision, improve patient outcomes, and streamline surgical workflows. Below are some key trends and potential future developments in the field. Advanced algorithms that involve segmentation of the spine and nonrigid deformations are currently under investigation [105]; however, as far as the authors are aware, these algorithms are not yet commercially available. Aubin et al., developed a preoperative planning simulator that incorporates patient-specific biomedical modeling of the spine to enable surgeons to predict scoliotic spine corrections based on instrumentation parameters [106]. Majdouline et al., used biomechanical modeling to demonstrate how instrumentation configurations can be optimized to achieve different clinical goals [107]. Incorporating patient-specific modeling into navigation solutions may eventually lead to improved surgical outcomes, particularly for long constructs. Furthermore, simpler navigation solutions based on patient-specific templates using 3D printing technologies have been successfully demonstrated for screw guidance [108].

Integration with robotic systems: The integration of navigation systems with robotic technology is expected to revolutionize spine surgery. Robotic systems can offer unparalleled precision and control, assisting surgeons with complex and minimally invasive procedures. The combination of robotics and navigation can lead to more consistent and accurate placement of surgical instruments, potentially reducing operative times and complication rates [109]. AR and VR technologies hold great promise for enhancing surgical navigation. AR can overlay critical information directly onto the surgeon’s view of the surgical field, improving spatial awareness and precision. VR can be used for surgical training, allowing surgeons to practice complex procedures in a simulated environment before performing them on patients [110]. Early studies indicate that AR-assisted surgeries can improve the accuracy and efficiency of surgical tasks, making it a valuable tool for the future of spine surgery [25].

The incorporation of intraoperative nerve integrity monitoring (NIM) into certain navigated spine instruments is a new development that offers additional safety assurance. NIM provides real-time feedback on the proximity of a stimulating probe or any other compatible instrumentation to a nerve root, thereby helping to detect pedicle breaches. This feature has been implemented in some navigated spine instruments, as noted in a study by Devlin et al. [111].

Finally, image quality improvement and decreased radiation dose are also important fields that should be focused on in future advancements. One research team has demonstrated that with algorithm improvements, it is possible to achieve comparable spine images at half of the radiation dose. The authors anticipate that future enhancements in hardware, algorithm design, and procedural workflow will result in additional dose reduction [99].

### 4.4. Strengths and Limitations

This narrative review provided a comprehensive and updated approach to the knowledge and implementation of modern navigated systems in spinal surgery and how such systems are utilized in all lumbar spinal surgery fields. One of the limitations is this study was concentrated on the lumbar spine. A more holistic assessment of navigation system including the cervical and thoracic spine should be included as a future succession of this study.

## 5. Conclusions

Advancements in navigation technologies have revolutionized lumbar spine surgeries, leading to safer and more effective procedures. Their initial limited utilization has further expanded to encompass almost all fields of lumbar spine surgeries. Future innovations are expected to further enhance the capabilities and accessibility of navigation systems, ultimately improving patient outcomes in lumbar spine surgery.

## Figures and Tables

**Table 1:** History of spine navigation.

Year	Trend	
1980s	Early CAS concepts	Initial ideas for computer-assisted surgery
1995	Image-guided surgery	First use in spine surgery with preoperative imaging
1999	Intraoperative imaging	Intraoperative use of CT scans, fluoroscopy, and ultrasonography
2000–2005	Optical tracking	Real-time instrument tracking systems
2006	Intraoperative 3D imaging	Registration fee
2010	Advanced navigation	Robotic assistance in 3D imaging
2011	Augmented reality (AR)	Spatial orientation improved
2015	Robotic assistance	Improved precision of procedures
2018	Artificial intelligence (AI)	Integrated image processing
2020	Mixed reality	To enable immersive planning and guidance

## Data Availability

The data presented in this study are available in the article.
